# Molecular Diagnostics Based on a Metabolite Risk Score Constructed Using Bilirubin and Oleic Acid in Korean Individuals with Obesity

**DOI:** 10.3390/biomedicines14030492

**Published:** 2026-02-24

**Authors:** Hye Jin Yoo

**Affiliations:** 1 Institute for Specialized Teaching and Research (INSTAR), Inha University, Incheon 22332, Republic of Korea; hyejin_yoo@inha.ac.kr; 2 BK21 FOUR Program in Biomedical Science and Engineering, Department of Biomedical Science, Inha University, Incheon 22332, Republic of Korea

**Keywords:** metabolite risk score, obesity, multi-biomarker-based molecular diagnostics, bilirubin, oleic acid

## Abstract

**Background/Objectives**: Obesity is currently diagnosed by anthropometric measures such as body mass index and waist circumference, while molecular-based diagnostic approaches have not yet been established. The present study aims to investigate the clinical meaning of a metabolite risk score (MRS) from the perspective of molecular diagnostics for obesity in Koreans. **Methods**: A total of 200 plasma samples were collected from 100 healthy individuals and 100 individuals with obesity through the Korea Biobank Network. Both groups were sub-divided into a discovery set and a validation set (*n* = 50 per group in each set). Metabolite profiling, cytokines, and oxidized (ox)-LDL were analyzed cross-sectionally. The MRS was constructed using statistically significant metabolites chosen based on linear regression analysis. **Results**: In the discovery set, systolic blood pressure, tumor necrosis factor-α, and interleukin-6 emerged as conventional obesity-related factors. Bilirubin and oleic acid were selected as key metabolites significantly associated with obesity. The MRS equation was constructed using these key metabolites through a weighted approach. In the validation set, the MRS demonstrated acceptable diagnostic accuracy for obesity (AUC = 0.748, *p* = 0.005). Furthermore, its diagnostic performance was enhanced when combined with the conventional obesity-related factors (AUC = 0.816, *p* < 0.001). **Conclusions**: The MRS, calculated using bilirubin and oleic acid, effectively complemented conventional obesity-related factors in the diagnosis of obesity. Although the MRS currently serves as a supportive molecular framework for obesity assessment, future refinement may improve its performance and broaden its applicability to other metabolic diseases.

## 1. Introduction

Obesity is a representative risk factor for various chronic diseases, such as hypertension, dyslipidemia, type 2 diabetes, cardiovascular diseases, and cancers. In addition, obesity itself is also considered a chronic disease. The most recent data, released in 2024, shows that the prevalence of obesity in Korean adults is 38.1%, and the rate has increased over the past decade [1]. Worldwide, the prevalence of obesity nearly tripled between 1975 and 2016 [2], and it is estimated that approximately half of the population will be overweight or obese by 2030 [3].

Obesity is a multifactorial disease influenced by both internal and external factors, including genetic predisposition, diet, and physical activity [4]. However, a common misconception is that obesity results from poor self-discipline [5], as it is often attributed to a lack of motivation to pursue physical activity and the greater availability of energy-dense foods. Excess accumulation of body fat is a typical trademark of obesity; thus, anthropometric measurements such as body mass index (BMI) and waist circumference are common and easy-to-use parameters to diagnose it [6]. Nevertheless, researchers have attempted to demonstrate that obesity and its complications can be diagnosed and prevented by molecules, including microRNAs, inflammatory biomarkers, adipocytokines, oxidative stress markers, and nutrients [7]. Based on this biological link between obesity and biomarkers at the molecular level, obesity is not caused by just a failure in self-management but also metabolic alterations that lead to excess fat accumulation and hinder weight loss [5].

Although molecular diagnostics for obesity are not currently used in clinical practice, review and research papers showed obesity-related changes in small molecules, called metabolites (<1500 Da), using metabolomics to understand the underlying mechanisms and metabolic characteristics of obesity [4,8,9,10]. Incidentally, most metabolomics studies have focused on overall metabolic pattern changes or finding significant individual metabolites. Few studies approach the concept of a metabolite risk score (MRS), which considers the comprehensive effects of biomarkers that show particular significance. So far, Ahola-Olli et al. [11] have constructed a weighted multi-metabolite score using phenylalanine, non-esterified cholesterol in large high-density lipoproteins (HDLs), and the cholesteryl ester-to-total-lipid ratio within large very-low-density lipoproteins (VLDLs) on type 2 diabetes in Finnish cohorts; He et al. [12] established a mild cognitive impairment MRS using 61 metabolites in the Hispanic/Latino population in the Study of Latinos-Investigation of Neurocognitive Aging (SOL-INCA) study; and finally, Geidenstam et al. [13] studied and validated an MRS using eight metabolites for predicting future weight gain in the Framingham Heart Study (FHS) and the Mexico City Diabetes Study (MCDS) cohorts. The studies concluded that the application of an MRS further enhanced the ability to predict these diseases. Therefore, constructing an MRS for obesity in a Korean population is a worthwhile endeavor. This can be achieved by discovering novel metabolites through ultra-performance liquid chromatography–tandem mass spectrometry (UPLC-MS/MS). Such an approach would facilitate the development of a molecular diagnosis strategy for obesity, based on a weighted multi-biomarker framework.

The aim of this study was to construct and validate an MRS for obesity in a Korean population using UPLC-MS/MS, a promising tool for non-targeted metabolomics that can detect a wide range of metabolites in a biospecimen. After profiling and screening significant obesity-associated metabolites, the MRS was constructed with a weighted approach that applied standardized β-coefficient values obtained from a linear regression analysis for each significant biomarker; then, the MRS was validated on another independent data set. This framework may improve the precision of obesity diagnosis in the context of personalized medicine by overcoming the limitations of anthropometric measures such as BMI, the only standardized diagnostic criterion, which is effective for population-level screening and epidemiological assessment yet limited in its utility for individualized clinical evaluation [14,15,16].

## 2. Materials and Methods

### 2.1. Samples and Dataset Acquisition

A total of 200 fasting plasma samples and their matched dataset used in this study were provided by the Biobank of Korea-Chungbuk National University Hospital (CBNUH) (Cheongju-si, Chungcheonghbuk-do, Republic of Korea) and the Biobank of Ajou University Hospital (Suwon-si, Gyeonggi-do, Republic of Korea), which are members of Korea Biobank Network, with the study protocol approved by the Institutional Review Board (IRB) of Inha University (approved IRB No.: 21004-1AR), being conducted in accordance with the Declaration of Helsinki.

Among the 200 samples, 100 samples were from individuals with obesity (obesity group), and the remaining were from individuals without any diseases (healthy group). They were randomly selected by the Biobanks according to the corresponding KCD codes [obesity group: Korean Standard Classification of Diseases (KCD) code, E66; healthy group: KCD code, Z00]. All of them were Korean adults (male or female) aged between 40 and 60. Subsequently, each group (*n* = 100) was randomly split into a discovery set and a validation set (1:1), with 50 subjects per group in each set. In the discovery set, key metabolite identification and MRS construction were performed, and in the validation set, the MRS was assessed.

### 2.2. Cytokines and Oxidized (Ox-) Low-Density Lipoprotein (LDL) Analysis

There were no data about inflammation and oxidative stress in the acquired dataset; analyses of cytokines and ox-LDL were carried out. Cytokines including interleukin (IL)-1β, IL-6, tumor necrosis factor (TNF)-α, interferon (IFN-γ) were analyzed by commercial kits, Human High Sensitivity T cell Magnetic Bead Panel (Merck Millipore, Billerica, MA, USA), according to the manufacturer’s instructions, and the resulting reactions were analyzed by a MAGPIX system (Luminex Corporation, Austin, TX, USA). Ox-LDL was analyzed using Ox-LDL kits (Mercodia, Uppsala, Sweden) based on the manufacturer’s instructions, and the resulting reactions were analyzed using SpectraMax 190 (Molecular Devices Corp., Sunnyvale, CA, USA).

### 2.3. Non-Targeted Metabolomics

#### 2.3.1. Sample Preparation

A total of 100 μL of fasting plasma samples was aliquoted and used for the UPLC-MS/MS analysis. A total of 300 μL of 70% cold acetonitrile (Thermo Fisher Scientific, Fair Lawn, NJ, USA) was added to the plasma samples. The mixtures were then incubated at 4 °C for 10 min after sufficient vortexing. Following incubation, the mixtures were centrifuged (13,000 rpm, 15 min, 4 °C), and the supernatant from each mixture was collected in a newly prepared microtube. The supernatant was dried using SpeedVac (Gyrozen, Gyeonggi-do, Republic of Korea); then, residues were re-dissolved in 10% methanol (J.T.Baker^®^; Avantor, Radnor, PA, USA) that contained internal standards (ISTD), L-leucine-^13^C_6_ (Sigma-Aldrich, St. Louis, MO, USA) and stearic-d_35_ acid (Sigma-Aldrich, St. Louis, MO, USA). For quality control (QC) samples, all plasma samples were pooled and prepared with the same procedure described above.

#### 2.3.2. Metabolite Analysis Using UPLC-MS/MS and Identification

Each prepared sample was injected into an Acquity UPLC-BEC-C18 column (2.1 mm × 100 mm, 1.7 μm; Waters, Milford, MA, USA) mounted in an Ultimate 3000 RSLC System (Thermo Fisher Scientific, Bremen, Germany) with a volume of 5 μL. QC samples were injected every 10th prepared plasma sample, also with a volume of 5 μL. The column temperature was kept constant at 50 °C. LC-MS grade water (Thermo Fisher Scientific, Fair Lawn, NJ, USA) and LC-MS grade acetonitrile (Thermo Fisher Scientific, Fair Lawn, NJ, USA), each containing 0.1% formic acid (Merck Millipore, Billerica, MA, USA), were employed as mobile phase A and B, respectively. Gradient changes ranging from 0% to 100% were applied over a period of 17 min at a flow rate of 0.4 mL/min. A Q-Exactive Orbitrap Plus MS (Thermo Fisher Scientific, Waltham, MA, USA) was utilized for MS analysis with both positive and negative electrospray ionization (ESI) modes. The scan type used was full MS-ddms^2^, covering a scan range of 80~1000 mass-to-charge (m/z). The MS conditions were as follows: spray voltage (kV), 3.5; sheath gas flow rate, 50 arbitrary units; auxiliary gas, 13 arbitrary units; S-lens radio frequency level, 55; and capillary temperature, 370 °C.

Detailed information on putative identification of metabolites was previously published [17]. Briefly, putative metabolite identification and relative peak intensity data were obtained via Compound Discoverer ver. 3.3 SP 2 software (Thermo Fisher, Waltham, MA, USA). The spectra were normalized based on the QCs, and the spectral features were annotated based on the ChemSpider and mzCloud databases integrated within the Compound Discoverer software.

### 2.4. The MRS Construction

#### 2.4.1. Discovery of Significant Obesity-Related Metabolites in the Discovery Set

From all identified metabolites, major metabolites were selected following the criteria: false discovery rate (FDR)-adjusted *p*-value (*q*-value) less than 0.05 and variable importance in projection (VIP) value over 1.5 (*q* < 0.05 and VIP ≥ 1.5). A linear regression analysis with a stepwise method of the obesity group on the major metabolites was performed to identify obesity-related metabolites. Finally, a receiver operating characteristic (ROC) curve was plotted to confirm which metabolites possessed suitable diagnostic ability for obesity. Subsequently, statistically significant metabolites identified in the ROC curve analysis were determined to be key metabolites for obesity.

#### 2.4.2. MRS Calculation

A linear regression analysis with an enter method was conducted again exclusively on the key metabolites for obesity to acquire standardized *β*-coefficient values in the discovery set. Then, the relative intensity values of each key metabolite were standardized to a *z*-score. Lastly, the equation for MRS was established as ∑*β_i_M_i_*, where *β_i_* represents the standardized *β*-coefficient value for each key metabolite utilized as a weight, and *M_i_* denotes the *z*-score of each key metabolite. The standardized *β*-coefficients (weight) obtained in the discovery set were locked down and applied to the MRS calculation for the validation set.

### 2.5. Comparison of Diagnostic Abilities

The diagnostic ability of the MRS was evaluated through ROC curve analysis, comparing it to: (1) a conventional marker model, (2) individual key metabolites, and (3) a combined model incorporating both conventional markers and MRS. Model 1 consisted of systolic blood pressure (BP), TNF-α, and IL-6, all of which emerged as significant independent markers of obesity in the discovery set; these three markers were identified through linear regression analysis with a stepwise selection process applied to the significant general clinical/biochemical markers, including systolic BP, TNF-α, IL-1β, and IL-6. Model 3 included the components of Model 1 along with the MRS. In the validation set, ROC curve analysis to compare diagnostic abilities was conducted in the same manner.

### 2.6. Statistical Analysis

The IBM SPSS Statistics 28.0 (IBM corp., Armonk, NY, USA) was used for the following statistical analysis. For between-group comparisons, an independent *t*-test was employed. In this analysis, variables that did not conform to a normal distribution were log-transformed. Confounding factors including age, sex, body weight, and BMI were adjusted by analysis of covariance (ANCOVA). Pearson’s correlation analysis was conducted to confirm correlations between variables. Linear regression analysis was employed for the selection of key metabolites; the acquisition of standardized *β*-coefficient values for MRS calculations; and the construction of both conventional and combined models for obesity. Through ROC curve analysis, the diagnostic ability for obesity of both models, the MRS, and individual key metabolites was evaluated. For all analyses, a two-tailed *p*-value less than 0.05 was considered significant. In the case of metabolite comparisons, FDR-adjusted *p*-values (*q*-values) were computed via an R package (fdrtool ver. 1.2.18) to control for type 1 error, with *q* < 0.05 indicating statistical significance.

SIMCA 17 software (Sartorius-Umetrics, Göttingen, Germany) was utilized to conduct an orthogonal partial least squares-discriminant analysis (OPLS-DA), generating loading plots, permutation test plots, and VIP scores. The model quality was assessed based on *R*^2^*Y* and *Q*^2^*Y* values (both >0.5), indicating goodness of fit and predictive ability, respectively. The model validity was evaluated through a permutation test (100 times) to avoid the problem of overfitting; a valid model is indicated if permuted *R*^2^*Y* values consistently fall below the actual *R*^2^*Y* values, and if the intercept of a regression line connecting the permuted *Q*^2^*Y* values and actual *Q*^2^*Y* value is below 0.

## 3. Results

### 3.1. General Clinical/Biochemical Markers Between the Groups in the Discovery Set

As shown in Table 1, individuals in the obesity group were significantly older than those in the healthy group. The sex distribution differed between the groups, and body weight and BMI were higher in the obesity group than in the healthy group. These four variables were employed for adjustment as confounding factors. Systolic BP, LDL-cholesterol, total cholesterol, TNF-α, IL-1β, and IL-6 retained their statistical significance, while the significance of glucose and ALT disappeared following adjustment. Among the significant variables, LDL-cholesterol and total cholesterol levels unexpectedly decreased in the obesity group. Despite these reductions, LDL-cholesterol levels particularly exhibited significant positive correlations with ox-LDL levels in the datasets of the current study (Figure S1). In addition, systolic BP, TNF-α, IL-1β, and IL-6 levels increased in the obesity group as expected. Note that, given this unexpected trend in lipid level changes, only systolic BP, TNF-α, IL-1β, and IL-6 were further used as key conventional markers among the statistically significant variables to establish a conventional model for obesity.

### 3.2. Metabolic Differences Between the Groups of the Discovery Set and Identification of the Key Metabolites

#### 3.2.1. Group Discrimination in the Discovery Set

A total of 452 and 258 metabolites were detected in the ESI positive and negative modes, respectively. After removing unknown and drug-related metabolites, 128 metabolites in the ESI positive mode and 69 metabolites in the ESI negative mode remained as putatively identified metabolites. OPLS-DA analysis was conducted using the identified metabolites.

**Figure 1:**
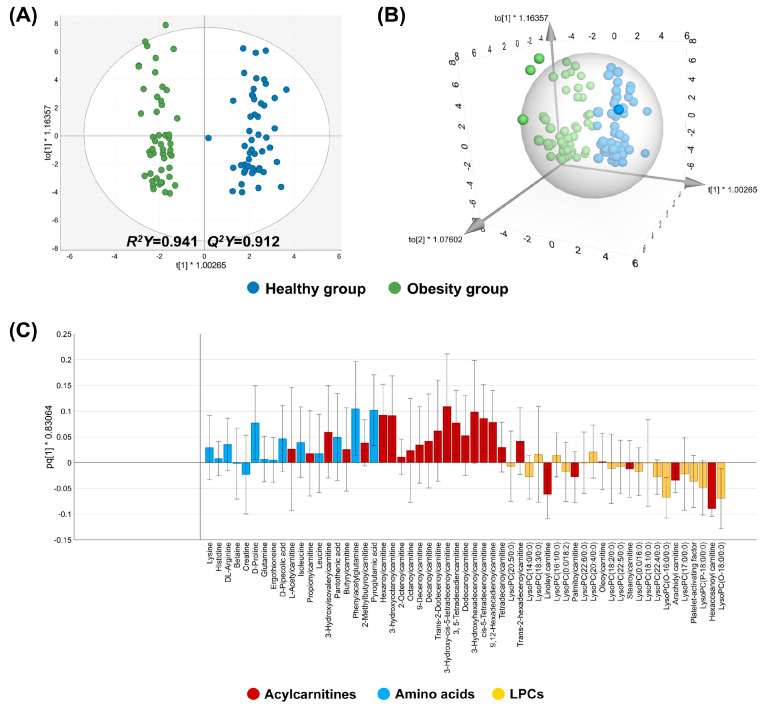
2D and 3D OPLS-DA and loading plots of the positive mode in the discovery set. (**A**,**B**) 2D and 3D OPLS-DA plots, respectively. (**C**) A loading plot of the top 3 abundant metabolite subclasses, including acylcarnitines, amino acids, and LPCs.

In the ESI positive mode, QC samples were tightly clustered (Figure S2A,B), and the healthy and obesity groups were effectively discriminated based on their metabolite characteristics, with high *R*^2^*Y* and *Q*^2^*Y* values (Figure 1A,B) and the permutation test results (Figure S3A). The results indicate that the OPLS-DA model displayed both good fit and predictive ability without an overfitting problem. For the loading plot analysis, metabolites belonging to subclasses such as acylcarnitines, amino acids, and lysophosphatidyl cholines (LPCs) were included, as these were the top three most abundant subclasses in the ESI positive mode (Table S1). Consequently, LPC metabolites were located on the side of the predictive component corresponding to the obesity cluster, whereas metabolites from the other subclasses were positioned toward the healthy side (Figure 1C).

**Figure 2:**
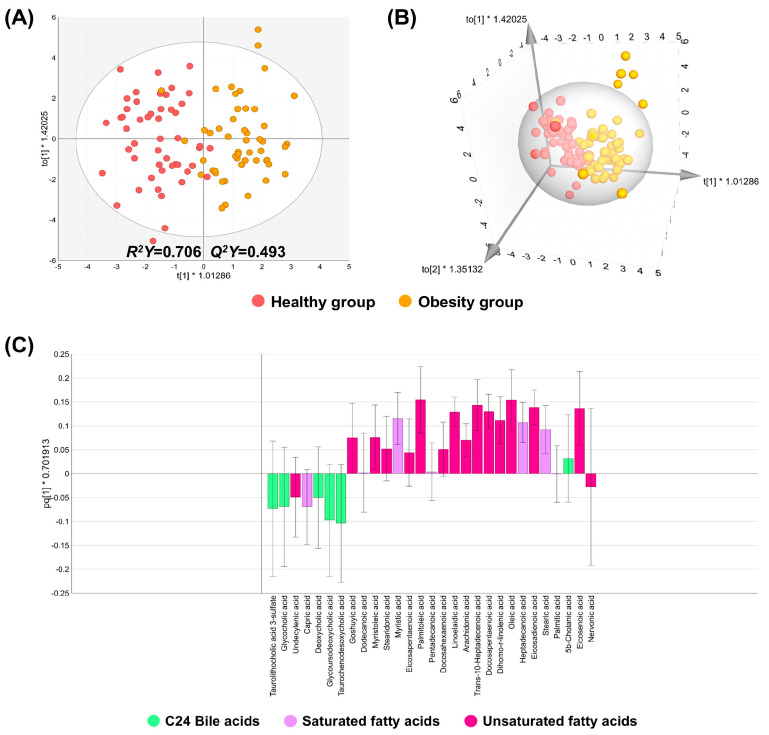
2D and 3D OPLS-DA and loading plots of the negative mode in the discovery set. (**A**,**B**) 2D and 3D OPLS-DA plots, respectively. (**C**) A loading plot of the top 3 abundant metabolite subclasses, including C24 bile acids, saturated fatty acids, and unsaturated fatty acids.

In the ESI negative mode, QC samples were also well clustered (Figure S2C,D). Between the healthy and obesity groups, the *R*^2^*Y* value was acceptable, whereas the *Q*^2^*Y* value fell just short of 0.5 by a very small margin, indicating that the model fit was good but the predictive ability was only 49.3% (*Q*^2^*Y* = 0.493) (Figure 2A,B). The result of the permutation test exhibited no evidence of an overfitting problem (Figure S3B). The top three abundant subclasses were selected for the loading plot analysis: unsaturated fatty acids (USFAs), saturated fatty acids (SFAs), and C24 bile acids (Table S1). The results showed that USFA and SFA metabolites were located on the obesity-associated side of the predictive component, whereas C24 bile acids were distributed towards the healthy-associated side (Figure 2C).

#### 3.2.2. Key Metabolite Selection

**Figure 3:**
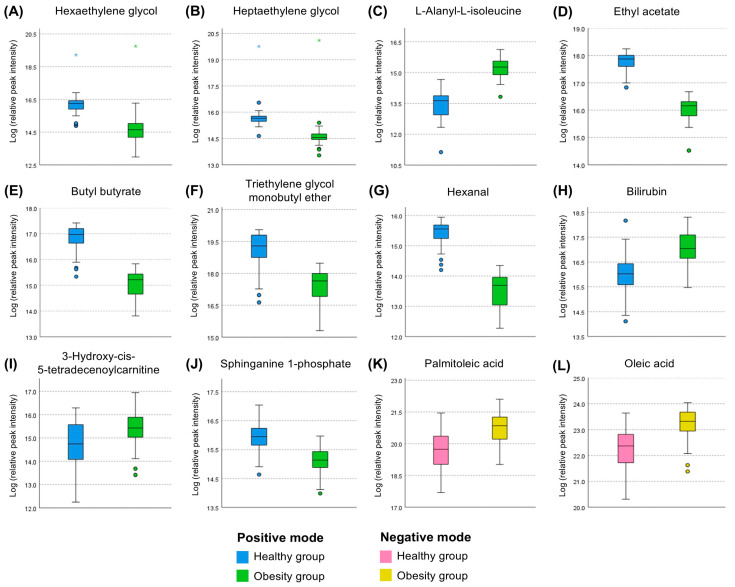
Box plots of the major metabolites in the positive and negative modes of the discovery set. Data are presented as median (interquartile range). Following logarithmic transformation, independent *t*-tests were performed to compare the healthy and obesity groups in the discovery set. The false discovery rate (FDR) was adjusted to present *q*-values for correcting multiple comparison error of metabolites; *q*-value < 0.05 was considered significant. (**A**–**J**) The major metabolites having both VIP score ≥ 1.5 and FDR-adjusted *q* < 0.05 in the positive mode of the discovery set. (**K**,**L**) The major metabolites having both VIP score ≥ 1.5 and *q* < 0.05 in the negative mode of the discovery set.

To discern major metabolites contributing to the discrimination between the two groups in the discovery set, VIP values were acquired (Figures S4 and S5). Following the criterion of a VIP value exceeding 1.5, 10 and 2 metabolites in the positive and negative modes, respectively, emerged as the major metabolites. Moreover, all of them showed statistical significance (*q^a^* < 0.001 and *q^b^* < 0.001) in the comparison between the healthy and obesity groups (Table S1, Figure 3). In the positive mode, among the 10 major metabolites, seven—hexaethylene glycol, heptaethylene glycol, ethyl acetate, butyl butyrate, triethylene glycol monobutyl ether, hexanal, and sphinganine 1-phospahte—showed decreased levels, while the remaining three—L-alanyl-L-isoleucine, bilirubin, and 3-hydroxy-cis-5-tetradecenoylcarnitine—exhibited increased levels. In the negative mode, all major metabolites, including palmitoleic acid and oleic acid, showed increased levels (Figure 3).

To figure out key metabolites significantly associated with obesity among the major metabolites, a linear regression analysis with a stepwise method was conducted. During the analysis, however, butyl butyrate and L-alanyl-L-isoleucine were excluded due to multicollinearity problems. Consequently, five metabolites, including ethyl acetate, triethylene glycol monobutyl ether, sphinganine 1-phosphate, bilirubin, and oleic acid, emerged as candidate key metabolites. ROC curve and logistic regression analyses were performed to evaluate the discriminatory performance and associations of these metabolites with obesity. As shown in Figure S6, ethyl acetate, triethylene glycol monobutyl ether, and sphinganine 1-phosphate showed high classification performance (Figure S6A). However, ethyl acetate and sphinganine 1-phosphate yielded unstable effect estimates in logistic regressions (Figure S6B), and triethylene glycol monobutyl ether was not considered clinically interpretable because of its limited biological relevance in the clinical context. Therefore, bilirubin and oleic acid, which showed good classification performance and stable effect estimates, were selected as the final key metabolites.

### 3.3. Calculation of MRS in the Discovery Set and Its Diagnostic Ability for Obesity

First of all, the relative peak intensities of the two key metabolites were converted into *z*-scores. Next, standardized *β*-coefficient values to be used as the weights were obtained via a linear regression model comprising the two key metabolites; the standardized *β*-coefficients of bilirubin and oleic acid were 0.416 and 0.421, respectively (Table 2). Lastly, the MRS was acquired following the equation of ∑*β_i_M_i_*, where *β_i_* and *M_i_* are the standardized *β*-coefficient value and the *z*-score of each metabolite, respectively.

**Figure 4:**
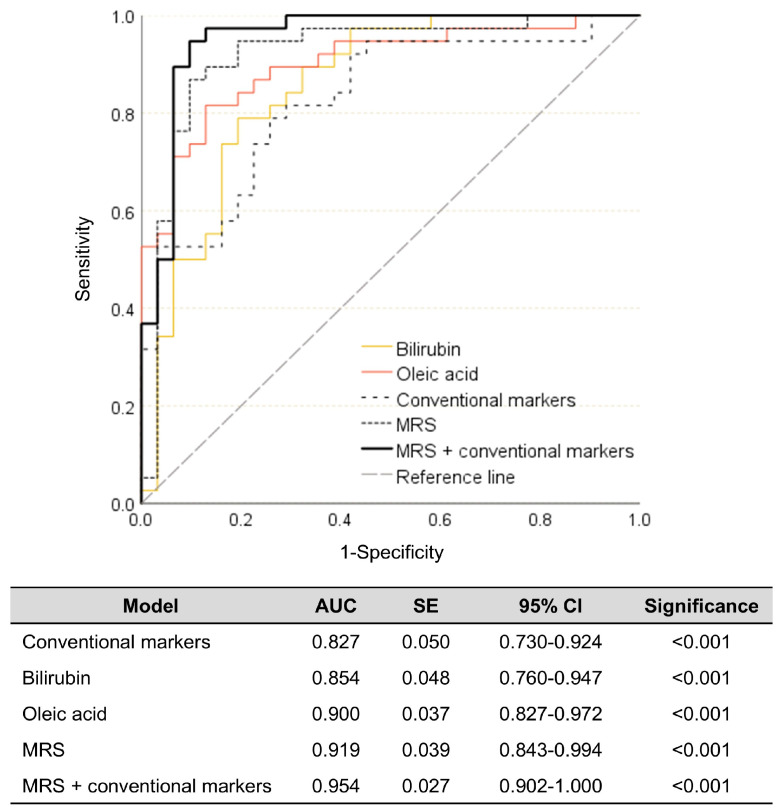
Diagnostic abilities of the two key metabolites, conventional markers, MRS, and combination of the MRS and conventional markers for obesity in the discovery set. Metabolite risk score (MRS) was calculated as ∑*β_i_M_i_*, where *β_i_* is a beta weight of a linear regression model comprising the two key metabolites (bilirubin and oleic acid), and *M_i_* is a *z*-score of the two key metabolites. A conventional marker model was established with systolic BP, TNF-α, and IL-6. AUC: area under the curve. CI: confidence interval. IL: interleukin. BP: blood pressure. SE: standard error. TNF-α: tumor necrosis factor-α.

Multi-diagnostic models for obesity were established, including those based on individual key metabolites, conventional markers, MRS, and an integrated approach combining conventional markers and MRS. As shown in Figure 4, the conventional model showed the lowest diagnostic ability for obesity (82.7%) among the models, but it was still a relatively high value and statistically significant. The bilirubin and oleic acid models displayed improved obesity diagnostic abilities (85.4% and 90.0%, respectively). Furthermore, the MRS model showed better enhanced diagnostic performance (91.9%). Finally, the combination of the MRS and conventional markers demonstrated a robust capability for obesity diagnosis, achieving an accuracy of 95.4% (Figure 4).

### 3.4. Verification of the MRS in the Validation Set

**Figure 5:**
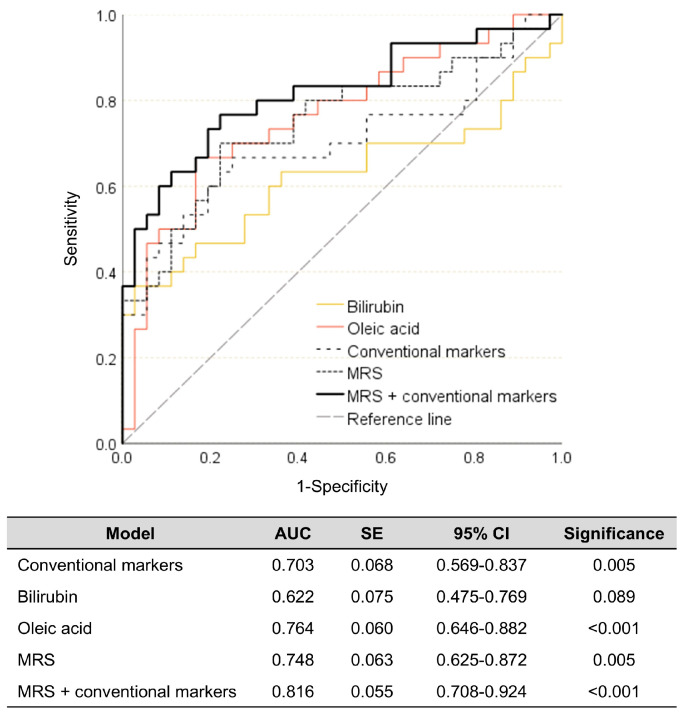
Diagnostic abilities of the two key metabolites, conventional markers, MRS, and combination of the MRS and conventional markers for obesity in the validation set. The metabolite risk score (MRS) was calculated as ∑*β_i_M_i_*, where *β_i_* is a beta weight of a linear regression model comprising the two key metabolites (bilirubin and oleic acid), and *M_i_* is a *z*-score of the two key metabolites. A conventional marker model was established with systolic BP, TNF-α, and IL-6. AUC: area under the curve. CI: confidence interval. IL: interleukin. BP: blood pressure. SE: standard error. TNF-α: tumor necrosis factor-α.

The MRS model derived from the discovery set was locked down and applied to the validation set, which was entirely independent from the discovery set. As illustrated in Figure 5, only the bilirubin model failed to demonstrate statistical significance and sufficient diagnostic ability (*p* = 0.089, 62.2%). In contrast to expectations, the obesity diagnostic ability of the MRS model (74.8%) was found to be lower than that of the key metabolite oleic acid (76.4%). This could be attributed to the low diagnostic ability of bilirubin, which may attenuate the overall diagnostic performance of the MRS model for obesity. Nevertheless, the MRS model exhibited improved diagnostic performance for obesity compared to the conventional model (70.3%). Lastly, the combination model consisting of the MRS and conventional markers exhibited the highest diagnostic capability for obesity in the validation set (81.6%), indicating that the use of the MRS along with conventional markers can enhance the ability to diagnose obesity (Figure 5).

## 4. Discussion

The MRS constructed in the present study is an identical concept to the genetic risk score (GRS). The GRS is the estimated overall probability or risk of a relevant disease in an individual based on their genetic background [18]; in other words, it is the cumulative effect of various genetic factors significantly involved in disease progression, thereby evaluating the contributions of multiple factors [18]. Indeed, many genetic loci interact with the development of certain diseases and their risk factors [18]. In the case of obesity, recent studies have tried to construct a GRS related to obesity using obesity-related genetic variants interacting with BMI and abdominal obesity [19,20]. As such, the GRS has been studied actively in the field of genomics, while there is a notable absence of approaches concerning MRSs in metabolomics, despite both belonging to the same omics field. Therefore, the present study tried to establish an MRS for obesity and assess its diagnostic ability for obesity using an independent validation set, since obesity is widely considered to be a risk factor for various diseases. Although further validation is required to establish the obesity MRS as a stand-alone molecular diagnostic tool, the findings suggest that the MRS may offer molecular-level discriminatory capacity for obesity, with bilirubin and oleic acid identified as its principal components.

Even though BMI, the only standardized method for obesity diagnosis, offers advantages such as cost-effectiveness, speed, and reproducibility for obesity diagnosis [14], its limitations have been discussed, as it does not directly account for body fat distribution and adipose tissue mass. For instance, BMI cannot determine abdominal (visceral fat) obesity, which poses greater health risks than non-abdominal (subcutaneous fat) obesity; BMI does not reflect age-related declines in muscle mass, which can result in a higher body fat proportion at a BMI below the obesity diagnostic cut-off (sarcopenic obesity); and BMI can misleadingly imply fat accumulation in individuals with high muscle mass and in patients with edema [6,14,15,16]. Moreover, the concept of metabolically healthy obesity has emerged, as some individuals who meet the obesity BMI cut-off present an apparently healthy phenotype [21]. In this regard, studies have sought to explore obesity from a molecular perspective, aiming to more precisely reflect the physiological responses and changes associated with obesity in order to identify potential biomarkers [6,22,23]. Despite the shift towards a personalized medical paradigm, the diagnosis of obesity is still dependent on anthropometric methods. To address the limitations of BMI and to more accurately identify individuals at high risk of obesity, the integration of molecular tools as an auxiliary method is necessary. In this context, the present study represents an effort demonstrating the potential of molecular diagnostics for obesity based on metabolomics through the introduction of the concept of the MRS.

Generally, obesity is accompanied by unfavorable lipid profiles. However, in the present study, both LDL-cholesterol and total cholesterol levels were unexpectedly found to be decreased. It is possible that these reduced cholesterol levels may be attributed to the use of prescription medications, as a total of 14 individuals in the obesity group were diagnosed with dyslipidemia. However, it is important to note that the present study did not collect medication history from the study subjects, so this remains speculative. Meanwhile, there are studies on lipid profile alteration in obesity that report findings consistent with those of the present study. One study divided 66 non-diabetic patients with obesity into three groups based on BMI, revealing that the highest-BMI group (class III obesity) exhibited significantly lower serum LDL-cholesterol and total cholesterol levels compared to the other two groups [24]. This difference was not attributable to lipid-lowering treatments, as the researchers found no statistically significant differences in the use of cholesterol-lowering medications (such as statin and ezetimibe) across the groups. Furthermore, the study demonstrated that BMI had significant negative correlations with both LDL-cholesterol and total cholesterol levels [24]. A recent LIPIDOGRAM 2004–2015 study also reported that the overweight group exhibited the highest levels of serum LDL-cholesterol and total cholesterol compared to other obesity groups [25]. These reductions have been supported by the following mechanisms: (1) the expansion of adipose tissue in obesity may enhance the activity of LDL receptors in adipose tissue, contributing to cholesterol reduction, and/or (2) a dilution effect on circulating LDL-cholesterol resulting from the elevated circulating blood volume commonly observed in obesity [24]. Consequently, the findings of the present study, along with the previous studies, suggest controversial aspects of lipid profiles in the context of obesity. Given this conflict, the inclusion of lipid levels may lead to inaccurate generalization; therefore, this issue must be carefully considered.

Meanwhile, despite the observed reduction, LDL-cholesterol levels exhibited significant positive correlations with the ox-LDL levels across all datasets in the present study (Figure S1). Although circulating LDL-cholesterol levels were lower in individuals with obesity compared to those who were healthy, these positive correlations may imply that elevated LDL-cholesterol levels in the context of obesity are more strongly correlated with oxidative stress. Oxidative stress is well known to induce pro-inflammatory responses in the body, and ox-LDL has been identified as one of the traditional risk factors for cardiovascular disease events, such as hypertension [26,27,28]. In this regard, the use of key conventional markers, including systolic BP, TNF-α, IL-1β, and IL-6, in the present study is deemed appropriate for the setup of a conventional model for obesity; ultimately, systolic BP, TNF-α, and IL-6 were selected for the final conventional obesity model in this study.

Bilirubin levels were significantly higher in the obesity group in the present study, which contrasts with previous reports describing inverse associations between bilirubin and obesity-related conditions, including metabolic syndrome, type 2 diabetes, and cardiovascular risks [29,30,31,32]. In contrast, a study showed increased bilirubin levels in individuals with obesity who exhibited a metabolically healthy phenotype [33]. These inconsistencies may reflect heterogeneity across study populations and clinical characteristics; therefore, the present findings should be interpreted with caution. One possible explanation is that bilirubin elevation may represent a compensatory response to inflammatory and oxidative stress, given its reported anti-inflammation and antioxidant properties [30]. Indeed, the obesity group in the present study showed significantly elevated levels of TNF-α, IL-1β, and IL-6, along with increased ox-LDL levels, although the latter did not reach statistical significance. Another possibility is that elevated bilirubin levels may be caused by hepatic damage [34]. Individuals with obesity often present higher levels of liver enzymes, AST and ALT, which are indicators of hepatocellular injury [34]. Indeed, in the present study, the obesity group showed increased AST and ALT levels beyond normal ranges (AST: 5 to 30 U/L; ALT: 4 to 36 U/L) [35], consistent with previous reports [36,37,38]. Importantly, logistic regression demonstrated that the association between bilirubin and obesity remained significant even after adjustment for AST and ALT levels (discovery set: *p* < 0.001, odds ratio = 7.794; validation set: *p* = 0.033, odds ratio = 2.021) in the present study. This suggests that bilirubin increases are not solely attributable to hepatic damage but may instead reflect broader metabolic alterations associated with obesity in the current study population. Therefore, while bilirubin may be considered a potential obesity-associated biomarker in this population, its directionality and underlying mechanisms should still be interpreted cautiously.

Plasma free fatty acids have been reported to increase in obesity [39]. Indeed, a total of 23 SFAs and USAFs were detected in the present study, and most of these showed elevated levels in the obesity group [fold change (obesity group to healthy group) ≥ 1.0, Table S1]. Particularly, oleic acid (C18:1), screened as a key metabolite, showed significant diagnostic ability for obesity in both our discovery and validation sets. Consistently, increased oleic acid levels were observed in individuals with obesity in the other research [40]. A study revealed that oleic acid promotes lipid accumulation via adipogenesis by upregulating the expression of CCAAT/enhancer binding protein α (C/EBPα) and peroxisome proliferator-activated receptor γ (PPARγ), which are adipogenic transcription factors [41]. This action of oleic acid implies that it could be regarded as a potential risk factor for obesity.

In addition to the key discriminatory metabolites selected in the present study, C24 bile acids have also been reported to be linked to the obesity phenotype. Cai et al. [42] showed that mice fed a high-fat diet for 16 weeks exhibited reduced circulating bile acid levels, which likely diminished activation of Takeda G protein-coupled receptor 5 (TGR5), a membrane receptor for bile acids. Attenuation of TGR5 signaling may disrupt energy expenditure and glucagon-like peptide-1 (GLP-1) secretion, thereby contributing to impaired lipid metabolism and obesity development. In the present study, the OPLS-DA loading plot in the ESI negative mode revealed that C24 bile acids were predominantly located on the healthy-associated side. This pattern suggests a relative shift away from the obesity cluster at the multivariate level. While the underlying mechanisms cannot be directly inferred, the collective metabolic signature captured in the multivariate model is directionally consistent with the previous findings linking altered bile acid levels to obesity-related metabolic imbalance.

There are limitations to this study. First, it proved challenging to get a dataset that fully met all requirements, such as the exclusion of medication usage and history of other diseases. Consequently, enrolling individuals exclusively with obesity was not easy. Despite that, this presented an opportunity for real-world validation by securing a cohort that closely resembled actual conditions. Second, further studies are needed to evaluate the MRS’s performance in larger cohorts. While the present sample size was sufficient to detect moderate-to-large metabolic differences and capture altered metabolic phenotyping [43,44], larger cohorts would allow more precise estimation of effect sizes and enhance the generalizability and stability of the identified metabolic signatures. Third, the present study adopted a straightforward weighted approach to identify independent obesity-related biomarkers. However, alternative modeling strategies, such as least absolute shrinkage and selection operator (LASSO), may further support the development of more refined MRS frameworks in future studies.

## 5. Conclusions

The aim of the present study was to construct an MRS using a weighted approach based on multiple significant obesity-associated metabolites and to evaluate its performance as a potential molecular diagnostic tool for obesity to overcome limitations of the current anthropometric diagnostic standard. Although the findings in this study do not support the immediate use of the MRS as a strong stand-alone molecular diagnostic tool, the results indicate that the MRS may provide additional discriminatory value when used alongside conventional obesity-related markers. In this context, the MRS may serve as a supportive molecular measure that captures metabolic alterations not fully reflected by anthropometric assessment alone.

Collectively, these findings suggest that the MRS framework, built with biologically meaningful biomarkers, offers a promising conceptual approach. Moreover, this weighted multi-biomarker strategy may be extendable to other metabolic or chronic diseases, supporting broader applications of molecular risk scoring in future diagnostic research.

## Figures and Tables

**Table 1:** General clinical/biochemical characteristics between the groups in the discovery set.

	Total (*n* = 100)				*p^a^*	*p^b^*
	Healthy (*n* = 50)		Obesity (*n* = 50)			
Age (year) *^†^*	45.6	±0.80	52.1	±0.66	<0.001	-
Male/Female *n*, (%)	35 (70.0)/15 (30.0)		44 (88.0)/6 (12.0)		0.027	-
Body weight (kg) *^†^*	68.4	±1.59	79.3	±1.59	<0.001	-
BMI (kg/m^2^)	23.8	±0.39	27.5	±0.39	<0.001	-
Systolic BP (mmHg) *^†^*	122.4	±1.63	137.9	±3.46	<0.001	0.006
Diastolic BP (mmHg)	78.1	±1.20	80.3	±2.37	0.398	0.252
Glucose (mg/dL) *^†^*	100.7	±1.53	109.0	±3.16	0.028	0.222
Triglyceride (mg/dL) *^†^*	146.5	±11.8	148.7	±12.5	0.915	0.427
HDL-cholesterol (mg/dL) *^†^*	50.2	±1.99	47.0	±2.01	0.198	0.756
LDL-cholesterol (mg/dL) *^†^*	112.9	±5.19	75.8	±5.18	<0.001	0.044
Total cholesterol (mg/dL) *^†^*	192.1	±4.89	143.82	±6.28	<0.001	<0.001
AST (U/L) *^†^*	23.6	±1.53	48.2	±22.6	0.197	0.938
ALT (U/L) *^†^*	24.4	±1.84	38.3	±5.29	0.024	0.603
TNF-α (pg/mL) *^†^*	9.13	±0.62	17.0	±1.65	<0.001	<0.001
IL-1β (pg/mL) *^†^*	3.74	±0.28	5.53	±0.53	0.009	0.006
IL-6 (pg/mL) *^†^*	2.68	±0.19	9.53	±4.75	0.006	0.016
IFN-γ (pg/mL) *^†^*	15.9	±1.26	14.3	±1.25	0.464	0.335
Ox-LDL (U/L)	49.9	±1.99	51.7	±3.00	0.610	0.564

Mean ± standard error (SE). *^†^* Variables were tested following logarithmic transformation. *p^a^*-values of the continuous variables were derived from independent *t*-tests. The *p^a^*-value of the sex distribution was derived from the chi-squared test. *p^b^*-values were *p^a^*-values that were adjusted by confounding factors, including age, sex, body weight, and BMI. All *p* < 0.05 were considered to indicate significance. ALT: alanine aminotransferase. AST: aspartate aminotransferase. BMI: body mass index. BP: blood pressure. HDL: high-density lipoprotein. IFN: interferon. IL: interleukin. LDL: low-density lipoprotein. Ox-: oxidized. TNF: tumor necrosis factor.

**Table 2:** Standardized *β*-coefficients of key metabolites, bilirubin and oleic acid, derived from linear regression analysis in the discovery set for obesity.

Independent Variables	Standardized *β*	*p*	CI	Significance *F*
Bilirubin	0.416	<0.001	0.152–0.336	<0.001
Oleic acid	0.421	<0.001	0.159–0.347	

CI: confidence interval.

## Data Availability

The data presented in this study are available on request from the corresponding author, as permission from the biobanks is required.

## Supplementary Materials

The following supporting information can be downloaded at https://www.mdpi.com/article/10.3390/biomedicines14030492/s1, Figure S1: Positive correlations between LDL-cholesterol and oxidized LDL, and decreased LDL-cholesterol levels in the obesity population across all datasets; Figure S2: 2D and 3D OPLS-DA plots incorporating the QC samples within the discovery set; Figure S3: Permutation tests for the OPLS-DA model of positive and negative modes in the discovery set; Figure S4: A VIP score plot of the top 50 metabolites observed in the positive mode in the discovery set; Figure S5: A VIP score plot of the top 50 metabolites observed in the negative mode in the discovery set; Figure S6: Selection of key metabolites associated with obesity based on ROC curve and logistic regression analysis; Table S1: Putative identification of plasma metabolites in the discovery set.

## Abbreviations

The following abbreviations are used in this manuscript:

ALT	Alanine aminotransferase
AST	Aspartate aminotransferase
BMI	Body mass index
BP	Blood pressure
ESI	Electrospray ionization
FDR	False discovery rate
GRS	Genetic risk score
HDL	High-density lipoprotein
IFN	Interferon
IL	Interleukin
ISTD	Internal standard
KCD	Korean standard classification of diseases
LDL	Low-density lipoprotein
LPC	Lysophosphatidyl choline
MRS	Metabolite risk score
OPLS-DA	Orthogonal partial least squares-discriminant analysis
Ox-	Oxidized
QC	Quality control
ROC	Receiver operating characteristic
SFA	Saturated fatty acid
TNF	Tumor necrosis factor
UPLC-MS/MS	Ultra-performance liquid chromatography-tandem mass spectrometry
USFA	Unsaturated fatty acid
VIP	Variable importance in projection
VLDL	Very-low-density lipoprotein
