# Supplementary material for: Molecular Diagnostics Based on a Metabolite Risk Score Constructed Using Bilirubin and Oleic Acid in Korean Individuals with Obesity
Source: Biomedicines. 2026 Feb 24;14(3):492. doi: 10.3390/biomedicines14030492 (PMC13024027; doi:10.3390/biomedicines14030492)
Supplement: Supplementary file 1 [file biomedicines-14-00492-s001.zip › biomedicines-4139400-supplementary.pdf]

Table S1. Putative identification of plasma metabolites in the discovery set

m/z	Subclass	Putative identification	VIP<br>Healthy *vs.* Obesity	q^a	q^b	Fold change
**Positive ion mode [M+H; ESI positive]**						
204.1228	Acyl carnitines	Acetylcarnitine	1.482	0.422	0.471	1.397
218.1384	Acyl carnitines	Propionylcarnitine	1.059	0.531	0.978	1.156
232.1542	Acyl carnitines	Butyrylcarnitine	1.008	0.467	0.754	1.183
246.1699	Acyl carnitines	2-Methylbutyroylcarnitine	0.451	0.395	0.752	1.013
260.1855	Acyl carnitines	Hexanoylcarnitine	1.197	0.002	0.008	1.339
262.1647	Acyl carnitines	3-Hydroxyisovalerylcarnitine	1.232	0.189	0.334	1.276
286.2012	Acyl carnitines	2-Octenoylcarnitine	0.318	0.486	0.898	0.891
288.2169	Acyl carnitines	Octanoylcarnitine	0.943	0.466	0.729	0.908
304.2116	Acyl carnitines	3-Hydroxyoctanoylcarnitine	1.284	0.004	0.098	1.333
314.2321	Acyl carnitines	9-Decenoylcarnitine	0.838	0.379	0.404	0.975
316.2477	Acyl carnitines	Decanoylcarnitine	1.016	0.241	0.401	1.010
342.2632	Acyl carnitines	Trans-2-Dodecenoylcarnitine	1.058	0.060	0.241	1.186
344.2788	Acyl carnitines	Dodecanoylcarnitine	1.016	0.126	0.405	1.074
368.2789	Acyl carnitines	3, 5-Tetradecadiencarnitine	1.175	0.022	0.075	1.294
370.2946	Acyl carnitines	Cis-5-Tetradecenoylcarnitine	1.239	0.006	0.046	1.309
372.3101	Acyl carnitines	Tetradecanoylcarnitine	0.670	0.381	0.807	1.026
386.2895	Acyl carnitines	3-Hydroxy-cis-5-tetradecenoylcarnitine	**1.502**	<0.001	0.010	1.373
396.3102	Acyl carnitines	9,12-Hexadecadienoylcarnitine	1.105	0.008	0.046	1.329
398.3258	Acyl carnitines	Trans-2-Hexadecenoylcarnitine	0.834	0.186	0.405	1.056
400.3414	Acyl carnitines	Palmitoylcarnitine	0.613	0.007	0.054	0.802
414.3207	Acyl carnitines	3-Hydroxyhexadecenoylcarnitine	1.301	0.001	0.031	1.529
424.3413	Acyl carnitines	Linoleyl carnitine	0.857	<0.001	0.002	0.659
426.3570	Acyl carnitines	Oleoylcarnitine	0.658	0.402	0.759	0.900
428.3727	Acyl carnitines	Stearoylcarnitine	0.300	0.192	0.489	0.831
456.4040	Acyl carnitines	Arachidyl carnitine	0.386	0.054	0.277	0.726
540.4979	Acyl carnitines	Hexacosanoyl carnitine	1.043	<0.001	<0.001	0.574
130.1591	Amines	Diisopropylethylamine	0.329	0.178	0.801	0.879

116.0707	Amino acids	Proline	0.930	0.108	0.478	1.096
118.0863	Amino acids	Betaine	0.642	0.469	0.994	1.118
130.0499	Amino acids	Pyroglutamic acid	1.120	0.004	0.069	3.386
130.0862	Amino acids	Pipecolic acid	0.659	0.274	0.396	1.048
132.0767	Amino acids	Creatine	0.692	0.302	0.980	0.902
132.1018	Amino acids	Isoleucine	0.743	0.393	0.530	1.179
132.1018	Amino acids	Leucine	1.283	0.529	0.503	1.298
147.0763	Amino acids	Glutamine	0.521	0.490	0.719	1.050
147.1127	Amino acids	Lysine	0.727	0.470	0.399	1.135
156.0768	Amino acids	Histidine	0.468	0.469	0.883	0.973
175.1190	Amino acids	Arginine	0.631	0.363	0.353	1.147
220.1178	Amino acids	Pantothenic acid	1.329	0.344	0.390	1.180
230.0955	Amino acids	Ergothioneine	0.725	0.567	0.538	0.962
265.1181	Amino acids	Phenylacetylglutamine	1.206	0.009	0.038	4.438
197.1284	Amino acids, peptides, and analogues	Cyclo(L-prolyl-L-valyl)	0.480	0.246	0.441	1.213
203.1501	Amino acids, peptides, and analogues	Ethyl argininate	0.899	0.156	0.192	1.202
232.1542	Amino acids, peptides, and analogues	Tert-Butoxycarbonyl-L-leucine	1.003	0.212	0.414	1.232
340.2840	Amino acids, peptides, and analogues	N-Oleoylglycine	1.420	<0.001	<0.001	1.654
448.3414	Amino acids, peptides, and analogues	N-Stearoyl tyrosine	0.928	<0.001	0.001	0.662
136.0392	Benzoxazolones	2-Benzoxazolol	0.886	0.064	0.167	1.372
585.2705	Bilirubins	Bilirubin	**1.628**	<0.001	<0.001	2.007
363.2166	C21 Steroids	Cortisol	0.507	0.217	0.409	1.367
387.2524	C24 Bile acids	12α-hydroxy-3-oxocholadienic acid	1.015	0.007	0.402	1.880
565.4035	C40 isoprenoids	ε,ε-Carotene-3,3'-dione	0.560	0.326	0.413	1.023
89.0601	Carboxylic acids	Ethyl acetate	**2.488**	<0.001	<0.001	0.207
162.1123	Carnitines	Carnitine	0.851	0.497	0.835	1.027
344.3153	Ceramides	N-acetylsphinganine	0.700	0.388	0.986	1.018
104.1072	Cholines	Choline	0.627	0.443	0.948	1.051
133.0317	Dihydrothiophenes	3-Methylsulfolene	1.152	0.500	0.891	1.215
203.1390	Dipeptides	L-Alanyl-L-isoleucine	**2.408**	<0.001	<0.001	2.256
229.1544	Dipeptides	Leucylproline	1.177	0.051	0.205	1.882

247.1287	Dipeptides	Aspartyl-Leucine	1.339	0.517	0.752	1.332
263.1389	Dipeptides	Phenylalanylproline	0.629	0.181	0.240	1.313
288.1551	Dipeptides	Methyl N-acetyl-L-alanyl-L-alanyl-L-alaninate	0.611	0.030	0.178	0.875
352.1653	Dipeptides	Tryptophyl-Phenylalanine	0.320	0.553	0.958	1.298
255.2104	Diterpenoids	18-Nor-4(19),8,11,13-abietatetraene	0.795	0.025	0.261	1.196
207.1589	Ethers	Triethylene glycol monobutyl ether	**2.348**	<0.001	<0.001	0.221
422.3258	Fatty acid esters	α-Linolenylcarnitine	0.648	0.025	0.042	0.762
442.3520	Fatty acid esters	3-Hydroxyoctadecenoylcarnitine	1.020	0.008	0.176	1.280
444.3675	Fatty acid esters	12-Hydroxy-12-octadecanoylcarnitine	0.393	0.306	0.330	0.984
328.2477	Fatty acids and conjugates	(9E)-9-Nitrooctadecenoic Acid	0.680	0.306	0.887	1.007
101.0963	Fatty aldehydes	Hexanal	**2.592**	<0.001	<0.001	0.196
280.2631	Fatty amides	9,12-Octadecadienamide	0.735	0.201	0.242	1.949
84.0812	Hydropyridines	1-Piperideine	0.345	0.553	0.798	1.077
114.0663	Imidazolines	Creatinine	0.977	0.224	0.372	1.310
192.0653	Indoleacetic acids	5-Hydroxyindoleacetic acid	1.183	0.387	0.463	1.221
188.0705	Indoles	Trans-3-Indoleacrylic acid	1.140	0.575	0.735	1.158
468.3078	LPC	LysoPC(14:0/0:0)	0.632	0.005	0.020	0.760
482.3600	LPC	LysoPC(O-16:0/0:0)	0.804	<0.001	0.015	0.642
494.3234	LPC	LysoPC(16:1/0:0)	0.520	0.420	0.414	1.031
496.3389	LPC	LysoPC(0:0/16:0)	0.671	<0.001	0.002	0.845
508.3761	LPC	LysoPC(P-18:0/0:0)	0.803	<0.001	0.001	0.686
510.3548	LPC	LysoPC(17:0/0:0)	0.675	0.020	0.029	0.742
510.3916	LPC	LysoPC(O-18:0/0:0)	0.949	<0.001	<0.001	0.624
518.3236	LPC	LysoPC(18:3/0:0)	0.567	0.549	0.887	0.838
520.3390	LPC	LysoPC(0:0/18:2)	0.408	0.052	0.719	0.915
520.3390	LPC	LysoPC(18:2/0:0)	0.604	0.009	0.219	0.907
522.3544	LPC	LysoPC(18:1/0:0)	0.487	0.416	0.413	0.907
524.3704	LPC	Platelet-activating factor	0.641	0.001	0.026	0.771
542.3235	LPC	LysoPC(20:5/0:0)	0.439	0.326	0.079	1.142
544.3386	LPC	LysoPC(20:4/0:0)	0.544	0.538	0.891	1.237
568.3387	LPC	LysoPC(22:6/0:0)	0.462	0.208	0.244	1.085

570.3544	LPC	LysoPC(22:5/0:0)	0.467	0.093	0.052	1.120
572.3700	LPC	LysoPC(22:4/0:0)	0.522	0.030	0.301	0.916
438.2971	LPE	LypoPE(P-16:0/0:0)	1.046	<0.001	<0.001	0.629
454.2920	LPE	LysoPE(16:0/0:0)	0.566	0.004	0.013	0.872
478.2923	LPE	LysoPE(18:2/0:0)	0.588	0.018	0.413	0.780
482.3234	LPE	LysoPE(0:0/18:0)	0.574	0.003	0.036	0.667
500.2764	LPE	LypoPE(20:5/0:0)	0.600	0.564	0.179	1.250
502.2921	LPE	LysoPE(0:0/20:4)	0.470	0.494	0.592	1.204
508.3391	LPE	LypoPE(0:0/20:1)	0.407	0.052	0.032	0.952
526.2923	LPE	LysoPE(22:6/0:0)	0.962	<0.001	<0.001	0.648
185.1284	N-alkylpyrrolidines	N-Acetylisoputreanine-γ-lactam	1.400	0.027	0.096	1.574
300.2891	NAE	Palmitoylethanolamide	0.457	0.238	0.283	1.088
326.3048	NAE	Oleoylethanolamide	0.813	0.025	0.069	1.236
550.3856	O-PC	PC(O-18:1/2:0)	0.767	<0.001	<0.001	0.755
270.2788	Other carboxylic acids	Capsiamide	0.601	0.012	0.079	0.881
126.0914	Oxazoles	5-Butyloxazole	1.204	0.011	0.122	1.497
792.5526	PC	PC(15:0/22:6)	0.852	<0.001	0.010	0.570
758.5690	PE-NMe	PE-NMe(18:1/18:1)	0.603	<0.001	<0.001	0.802
107.0494	Phenolic acids	Benzaldehyde	0.710	0.296	0.245	1.185
123.0441	Phenolic acids	4-Hydroxybenzaldehyde	1.045	0.572	0.704	1.215
352.2238	Phosphate esters	Sphingosine 1-phosphate (d16:1-P)	0.716	0.004	0.195	0.690
703.5742	Phosphosphingolipids	SM(d18:1/16:0)	0.614	0.002	0.016	0.893
316.2840	Phytosphingosines	Dehydrophytosphingosine	0.232	0.476	0.678	0.992
86.0968	Piperidine alkaloids	Piperidine	0.642	0.427	0.719	1.151
286.1432	Piperidine alkaloids	Piperine	0.650	0.247	0.978	0.918
283.1750	Polyethylene glycols	Hexaethylene glycol	**2.078**	<0.001	<0.001	0.504
327.2010	Polyethylene glycols	Heptaethylene glycol	**1.554**	<0.001	<0.001	0.765
195.0876	Purines and purine derivatives	1,3,9-Trimethylxanthine	0.139	0.473	0.994	0.852
100.0760	Pyrrolidones	2-Pyrrolidinone	0.598	0.550	0.806	1.088
269.2260	Sesquiterpenoids	Anhydroretinol	0.232	0.383	0.747	1.082
145.1222	Short fatty esters	Butyl butyrate	**2.516**	<0.001	<0.001	0.208

146.0599	Simple indole alkaloids	1H-Indole-4-carbaldehyde	0.649	0.490	0.980	1.148
302.3048	Sphinganines	Sphinganine	1.471	<0.001	<0.001	0.426
380.2553	Sphingoid base 1-P	Sphingosine-1-phosphate	1.184	<0.001	<0.001	0.568
382.2709	Sphingoid base 1-P	Sphinganine 1-phosphate	**1.522**	<0.001	<0.001	0.452
300.2892	Sphingosines	Sphingosine	1.342	<0.001	<0.001	0.466
427.3567	Triterpenoids	4β-Methylzymosterol-4-carbaldehyde	0.875	0.005	0.036	1.352
181.0718	Xanthines	Theobromine	0.409	0.360	0.938	0.772
181.0719	Xanthines	Paraxanthine	0.582	0.501	0.634	1.129
**Negative ion mode [M-H; ESI negative]**						
178.0503	Acylaminobenzoic acids	4-Acetamidobenzoic acid	1.182	0.049	0.290	3.368
180.0660	Amino acids	Tyrosine	1.136	0.264	0.988	1.543
203.0822	Amino acids	Tryptophan	0.841	0.370	0.823	1.059
775.6817	Amino acids	Thyroxine	0.789	0.435	0.440	1.064
284.2236	Amino acids, peptides, and analogues	Myristoylglycine	0.169	<0.001	0.003	1.284
203.0015	Arylsulfates	O-Methoxycatechol-O-sulphate	1.026	0.306	0.835	1.514
247.1705	Benzene and substituted derivatives	[2-(Dimethoxymethyl)-1-heptenyl]benzene	0.896	0.014	0.244	1.227
193.1229	Benzenediols	Hexylresorcinol	0.717	0.374	0.556	0.958
172.9906	Benzenesulfonic acids	4-Hydroxybenzenesulfonic acid	0.958	0.438	0.879	1.178
311.1692	Benzenesulfonic acids	N-Undecylbenzenesulfonic acid	1.011	0.034	0.125	0.918
325.1848	Benzenesulfonic acids	4-Dodecylbenzenesulfonic acid	1.075	0.036	0.069	0.840
367.1590	C19 Steroids	Testosterone sulfate	1.068	0.083	0.597	1.124
369.1747	C19 Steroids	Androsterone sulfate	0.845	0.314	0.997	1.186
280.6225	C24 Bile acids	Taurolithocholic acid 3-sulfate	0.828	0.329	0.921	1.282
359.2961	C24 Bile acids	5β-Cholanic acid	0.435	0.011	0.035	1.445
391.2863	C24 Bile acids	Deoxycholic acid	0.776	0.320	0.836	1.076
448.3079	C24 Bile acids	Glycoursodeoxycholic acid	1.314	0.051	0.197	0.914
464.3027	C24 Bile acids	Glycocholic acid	1.158	0.197	0.474	1.127
498.2907	C24 Bile acids	Taurochenodesoxycholic acid	1.355	0.010	0.177	0.924
435.3488	C27 Bile acids	5β-cholestan-3α,7α,24,27-tetrol	0.682	0.060	0.003	1.473
357.2805	Carbonyl compounds	1-Phenyl-1,3-octadecanedione	0.956	0.002	0.110	1.888
433.2369	Carbonyl compounds	Oleoylglycerone phosphate	1.214	0.196	0.980	0.855

199.0066	Cinnamic acids	4-Vinylphenol sulfate	0.759	0.367	0.798	1.287
267.1242	Heterocyclic FA	3-carboxy-4-methyl-5-pentyl-2-furanpropanoic acid	0.459	0.055	0.019	1.441
299.2594	HODA	9-Hydroxyoctadecanoic acid	0.385	0.033	0.197	1.162
311.2234	HpODE	9-HPODE	0.416	0.007	0.002	1.420
204.0662	Indolecarboxylic acids	Indolelactic acid	0.757	0.119	0.730	1.170
277.2176	Lineolic acids and derivatives	α-Eleostearic acid	1.040	<0.001	0.006	1.595
409.2368	LPA	LysoPA(16:0/0:0)	1.378	0.026	0.320	0.770
435.2524	LPA	LysoPA(18:1/0:0)	1.268	0.054	0.413	0.843
437.2684	LPA	LysoPA(18:0/0:0)	1.232	0.001	0.001	0.770
463.2840	LPA	PA(20:1/0:0)	1.435	0.013	0.027	0.683
508.3418	LPE	LysoPE(20:0/0:0)	1.271	<0.001	<0.001	0.731
599.3216	LPI	LysoPI(18:0/0:0)	1.005	0.066	0.085	0.965
327.2546	MAG	MG(16:1/0:0/0:0)	0.634	0.400	0.195	1.395
201.0223	Other phenols	4-Ethylphenylsulfate	1.020	0.421	0.589	1.841
239.0674	Other phenols	2-(1-Methylpropyl)-4,6-dinitrophenol	0.980	0.229	0.898	0.955
738.5105	PE	PE(16:0/20:4)	1.137	0.002	0.011	0.744
187.0064	Phenylsulfates	p-Cresol sulfate	0.960	0.107	0.404	1.369
243.0623	Pyrimidine ribonucleosides	Uridine	0.676	0.232	0.798	1.025
171.1383	Saturated fatty acid	Capric acid	0.668	0.368	0.735	0.991
199.1699	Saturated fatty acid	Dodecanoic acid	0.335	0.071	0.347	1.049
227.2015	Saturated fatty acid	Myristic acid	1.197	<0.001	<0.001	1.548
241.2173	Saturated fatty acid	Pentadecanoic acid	0.245	0.003	0.003	1.180
255.2332	Saturated fatty acid	Palmitic acid	0.294	0.002	0.046	1.159
269.2491	Saturated fatty acid	Heptadecanoic acid	1.043	<0.001	<0.001	1.589
283.2646	Saturated fatty acid	Stearic acid	0.936	<0.001	<0.001	1.520
212.0019	Simple indole alkaloids	Indoxyl sulfate	1.011	0.192	0.458	1.966
465.2503	Steroidal glycosides	5α-Dihydrotestosterone glucuronide	1.030	0.029	0.118	4.077
191.0191	TCA acids	Isocitric acid	0.867	0.346	0.886	1.013
183.1384	Unsaturated fatty acid	Undecylenic acid	0.506	0.319	0.719	1.042
223.1702	Unsaturated fatty acid	Goshuyic acid	1.029	0.003	0.006	1.297
225.1858	Unsaturated fatty acid	Myristoleic acid	0.827	<0.001	0.001	1.409

253.2174	Unsaturated fatty acid	Palmitoleic acid	**1.600**	<0.001	<0.001	1.824
267.2334	Unsaturated fatty acid	Trans-10-Heptadecenoic acid	1.462	<0.001	<0.001	1.815
275.2022	Unsaturated fatty acid	Stearidonic acid	0.706	0.002	0.096	1.518
279.2332	Unsaturated fatty acid	Linoelaidic acid	1.325	<0.001	<0.001	1.741
281.2489	Unsaturated fatty acid	Oleic acid	**1.534**	<0.001	<0.001	1.862
301.2177	Unsaturated fatty acid	Eicosapentaenoic acid	0.556	<0.001	0.071	1.883
303.2333	Unsaturated fatty acid	Arachidonic acid	0.698	<0.001	<0.001	1.835
305.2490	Unsaturated fatty acid	Dihomo-γ-linolenic acid	1.136	<0.001	<0.001	1.860
307.2647	Unsaturated fatty acid	Eicosadienoic acid	1.396	<0.001	<0.001	1.841
309.2804	Unsaturated fatty acid	Eicosenoic acid	1.337	<0.001	<0.001	2.023
327.2334	Unsaturated fatty acid	Docosahexaenoic acid	0.769	<0.001	0.022	1.635
329.2491	Unsaturated fatty acid	Docosapentaenoic acid	1.389	<0.001	<0.001	2.077
365.3429	Unsaturated fatty acid	Nervonic acid	0.513	0.315	0.978	1.139
295.2646	Wax diesters	Methyl oleate	1.311	<0.001	<0.001	1.602
255.2331	Wax monoesters	Ethyl tetradecanoate	1.265	<0.001	<0.001	1.660
167.0203	Xanthines	Uric acid	0.833	0.437	0.983	1.034

The plasma metabolites were putatively identified. All variables tested following logarithmic transformation. q^a-Values are FDR-adjusted *p*-value which were derived from independent *t-tests*. q^b-Values are FDR-adjusted *p*-value derived from age- and sex-adjusted analysis of covariance (ANCOVA). FC values were calculated by dividing relative peak intensity values of the obesity group into those of the healthy group (obesity group/healthy group). ESI: electrospray ionization. FC: fold change. FDR: false discovery rate. VIP: variable importance in projection.

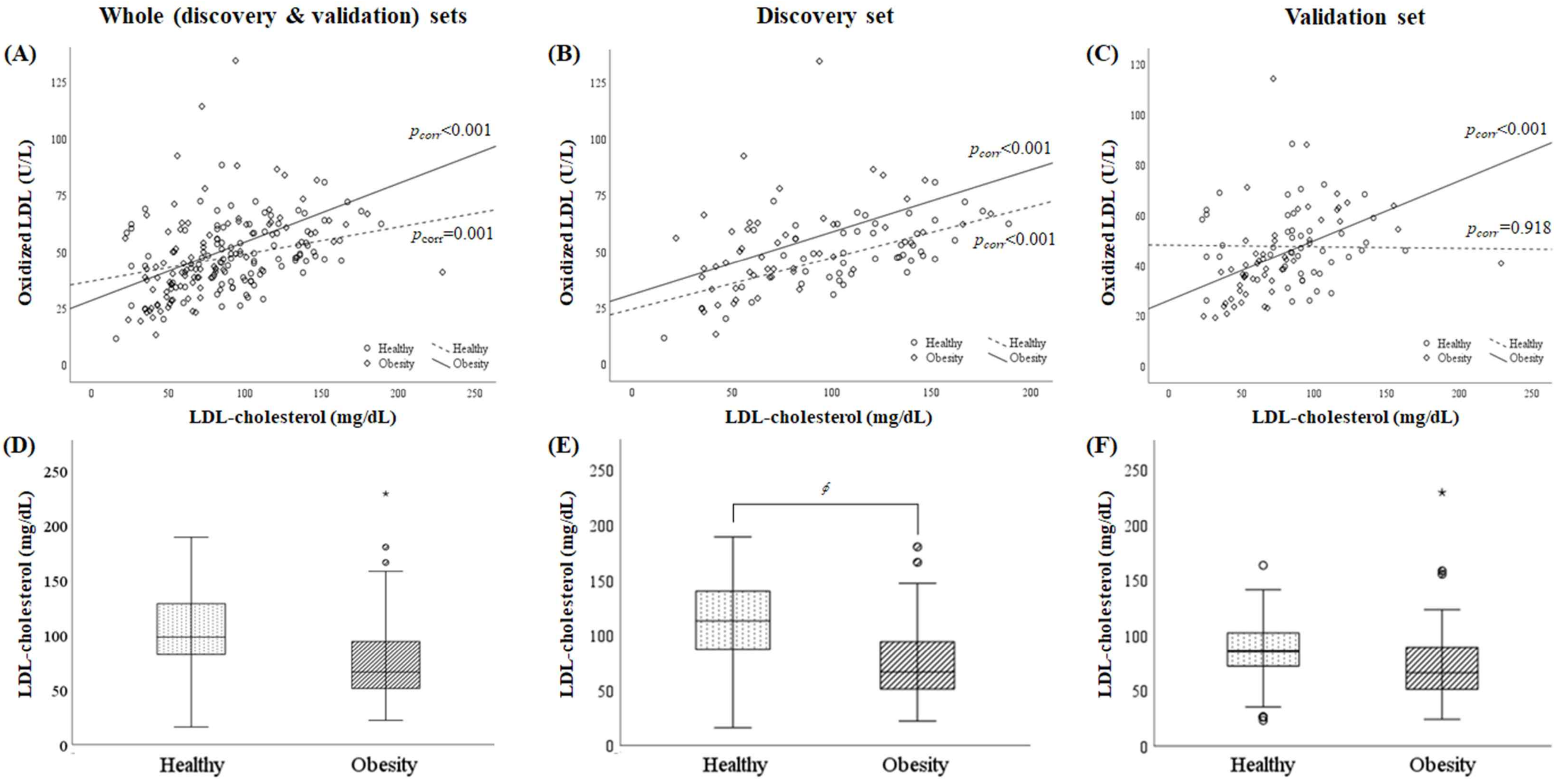

Figure S1. Positive correlations between LDL-cholesterol and oxidized LDL, and decreased LDL-cholesterol levels in the obesity population across all datasets

(A-C) LDL-cholesterol and oxidized LDL exhibited significant positive correlations in the obesity groups across all datasets, with *p*-values derived from *Pearson*'s correlation analysis.
(D-F) LDL-cholesterol levels were reduced in the obesity groups across all datasets; however, statistical significances was only observed in the discovery set (∮ *p*<0.05).

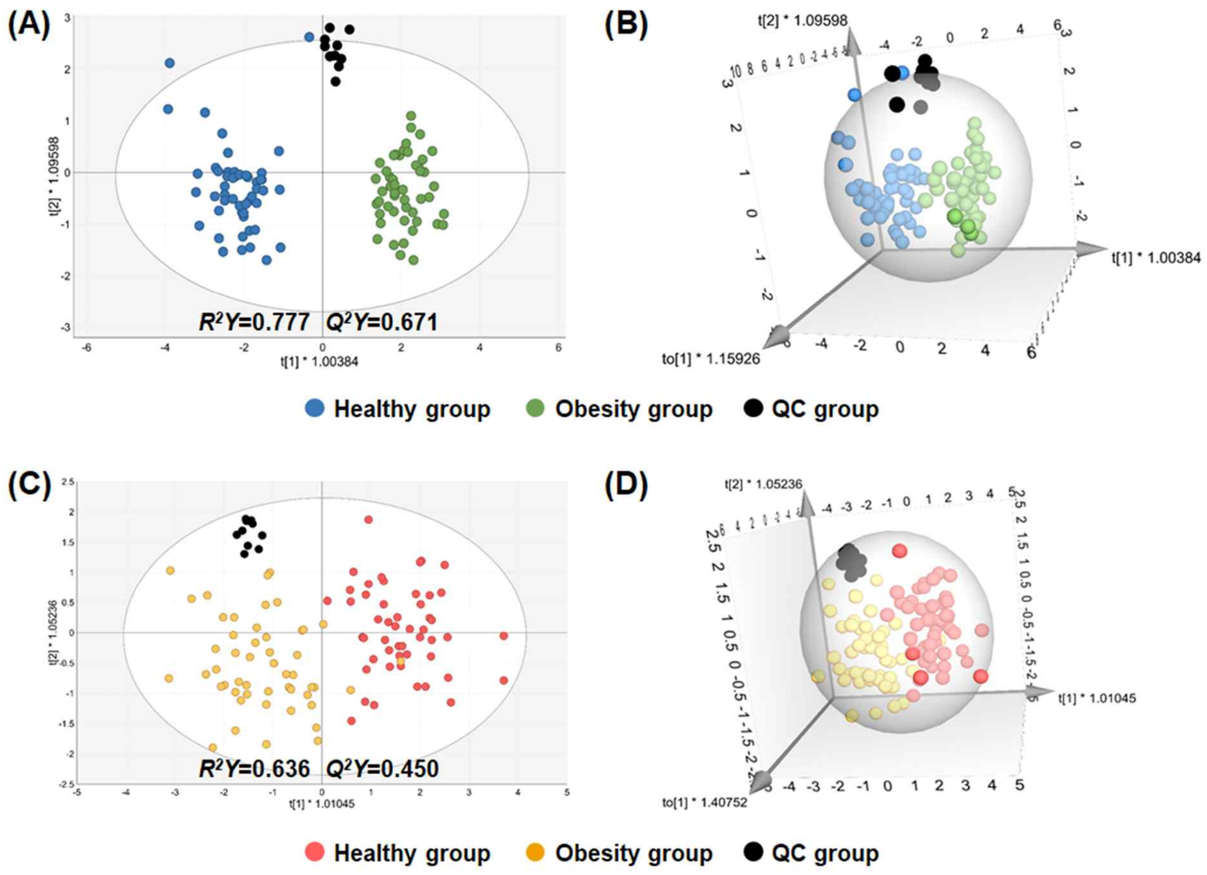

Figure S2. 2D and 3D OPLS-DA plots incorporating the QC samples within the discovery set

(A, B) The 2D and 3D OPLS-DA plots in the positive mode, respectively. **(C, D)** The 2D and 3D OPLS-DA plots in the negative mode, respectively.

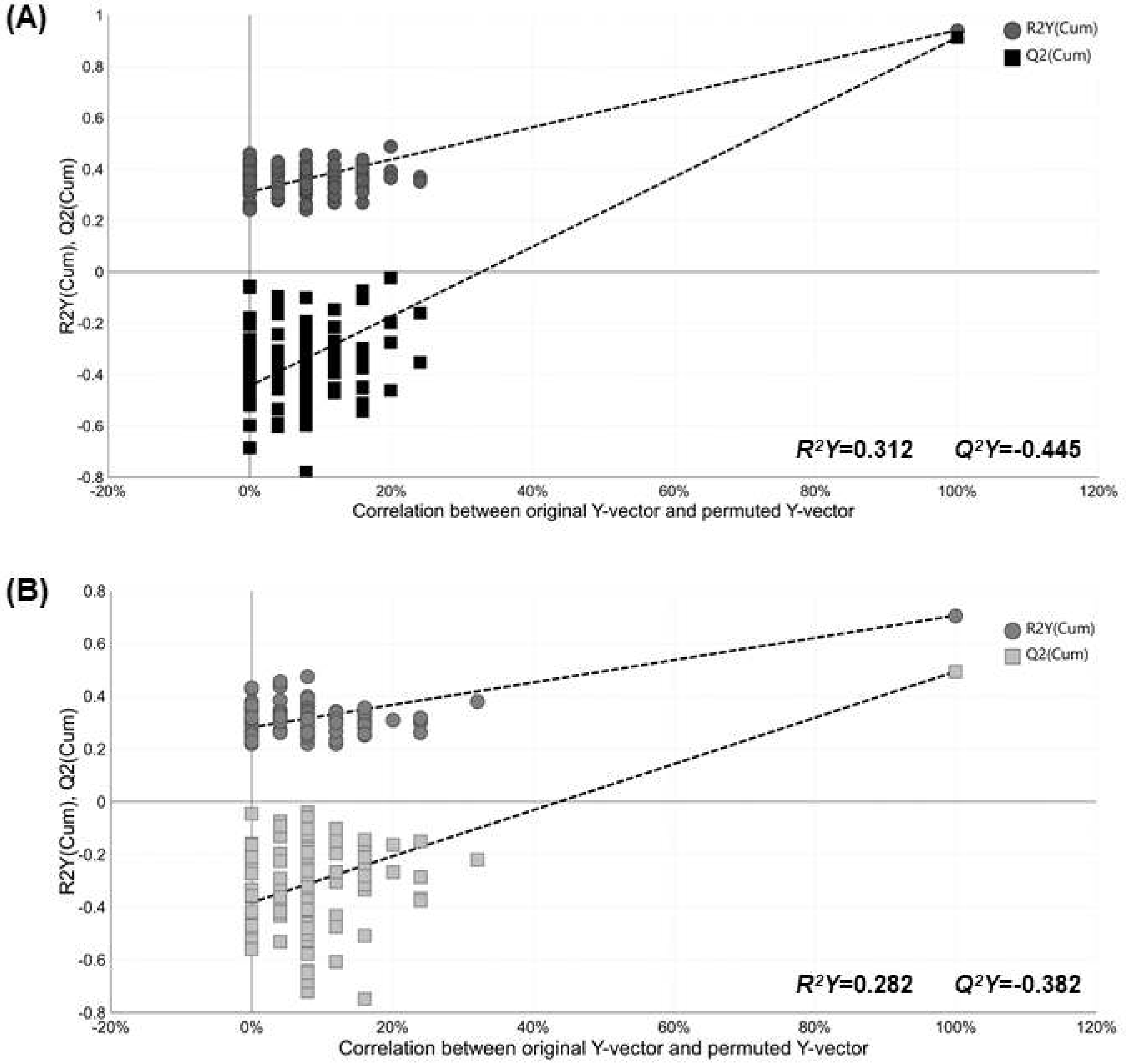

Figure S3. Permutation tests for the OPLS-DA model of positive and negative modes in the discovery set

100 permutation tests were performed. (A) The permutation test result of the OPLS-DA model of the positive mode in the discover set. (B) The permutation test result of the OPLS-DA model of the negative mode in the discovery set.

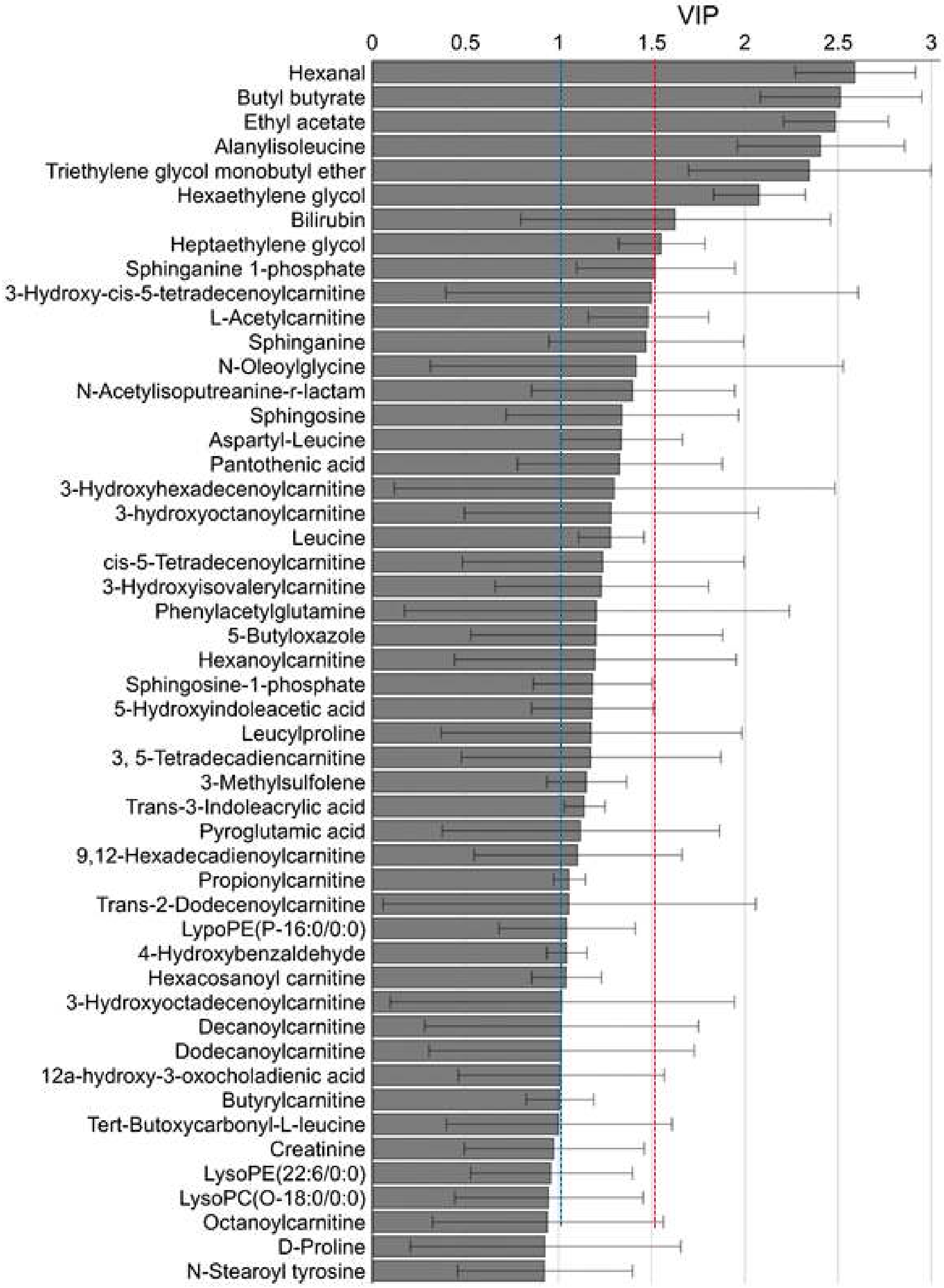

Figure S4. A VIP score plot of the top 50 metabolites observed in the positive mode in the discovery set

The blue and red lines indicate VIP scores of 1.0 and 1.5, respectively. A total of 44 metabolites have a VIP value over 1.0; among them, 10 metabolites have a VIP value over 1.5.

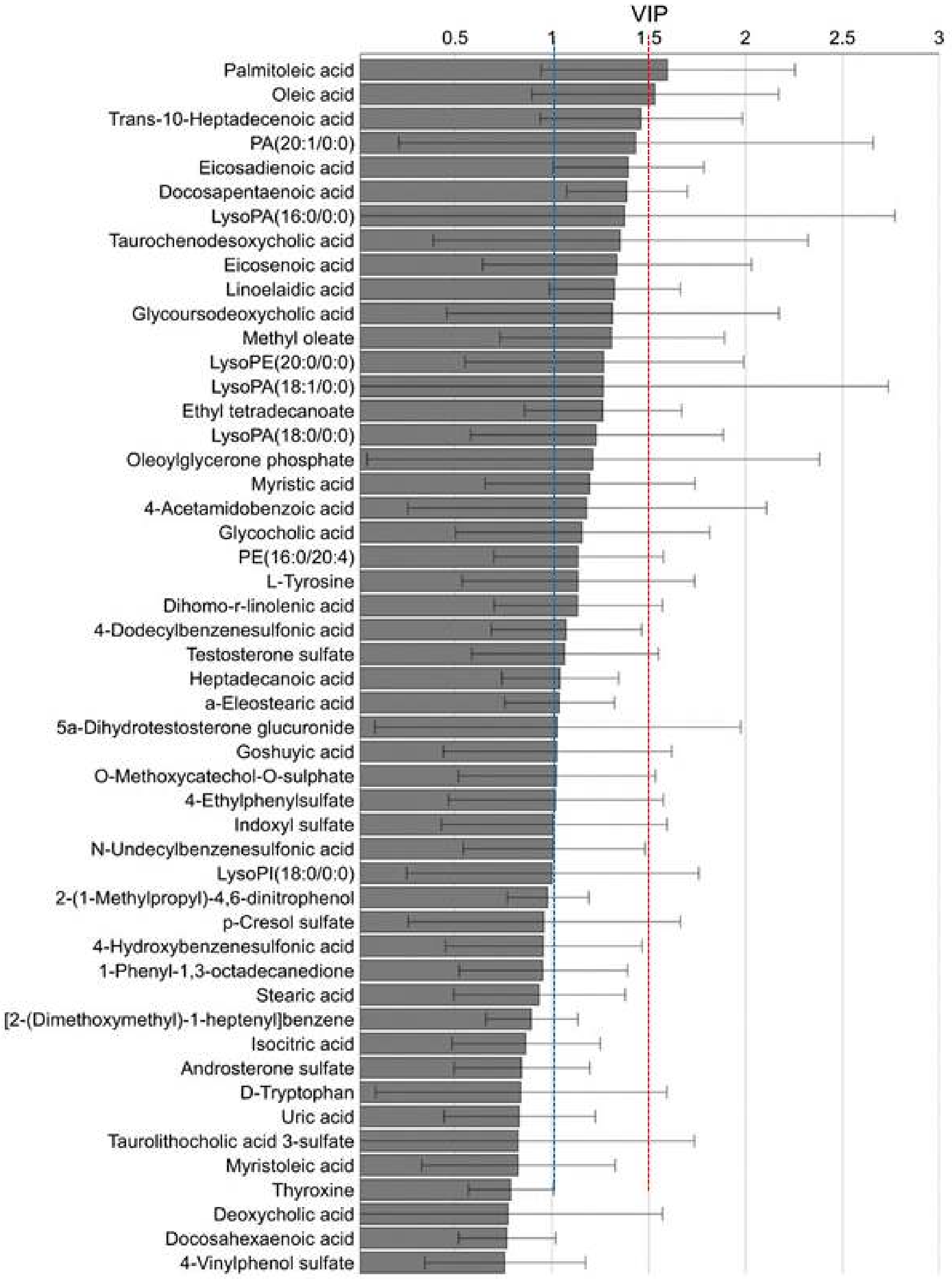

Figure S5. A VIP score plot of the top 50 metabolites observed in the negative mode in the discovery set

The blue and red lines indicate VIP scores of 1.0 and 1.5, respectively. A total of 34 metabolites have a VIP value over 1.0; among them, 2 metabolites have a VIP value over 1.5.

(A) ROC curves for the five candidate key metabolites

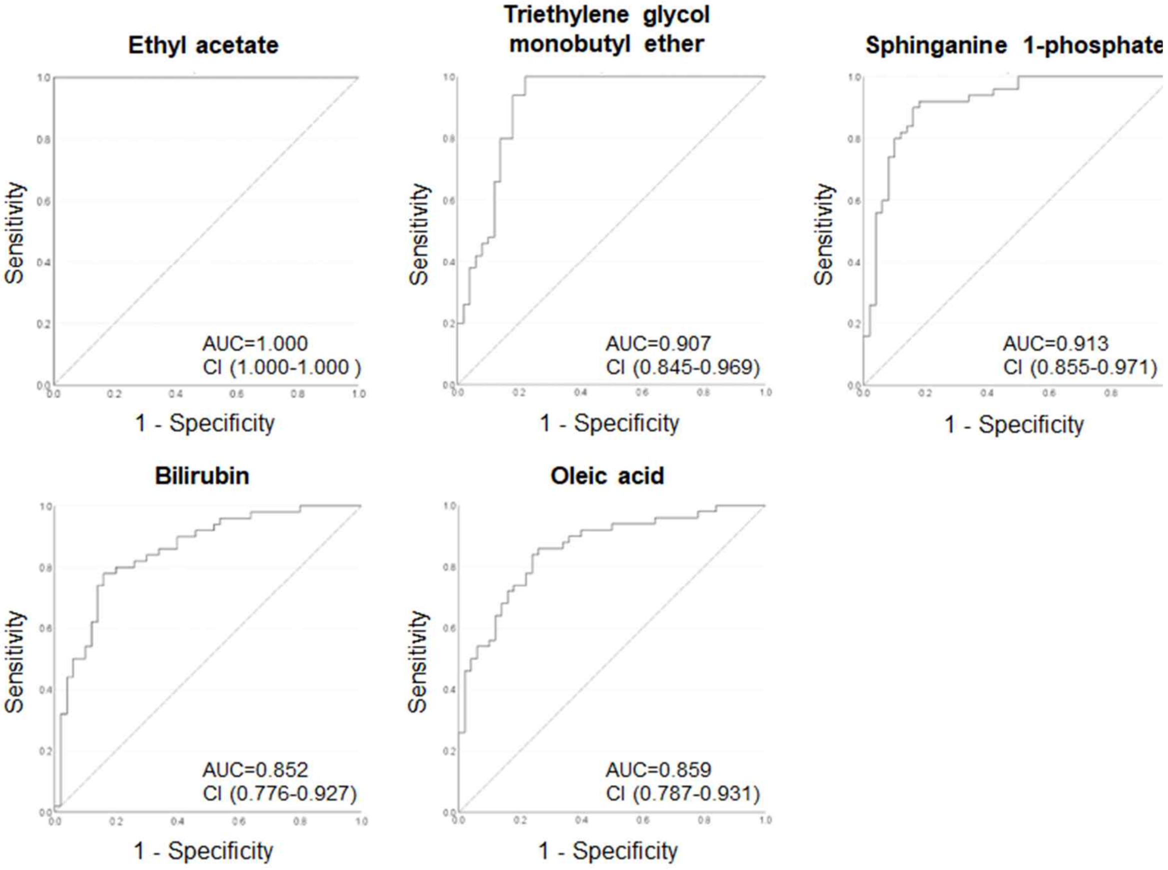

(B) Odds ratio for the five candidate key metabolites

Metabolites	*p*	Odds ratio	95% CI
Ethyl acetate	0.982	0.000	Not estimable
Triethylene glycol monobutyl ether	<0.001	0.104	0.044–0.247
Sphinganine 1-phosphate	<0.001	0.012	0.002–0.068
Bilirubin	<0.001	7.994	3.498–18.267
Oleic acid	<0.001	9.442	3.807–23.421

Figure S6. Selection of key metabolites associated with obesity based on ROC curve and logistic regression analyses

Each candidate metabolite was evaluated individually as a single predictor of obesity in the discovery set. (A) ROC curve analysis. (B) Logistic regression analysis. Metabolite levels were log-transformed prior to logistic regression analysis. Therefore, each odds ratio represents the change in obesity odds associated with a one-unit increase in the log-transformed metabolite level, corresponding to an approximately 2.7-fold increase in the original level.

Ethyl acetate showed non-estimable confidence intervals and unstable estimates in logistic regression, indicating potential model instability. Sphinganine 1-phosphate exhibited an extreme odds ratio, corresponding to an approximately 83-fold reduction in obesity odds, despite demonstrating good discriminatory performance in ROC curve analysis. Triethylene glycol monobutyl ether also exhibited good performance and a significant odds ratio; however, its biological relevance in the clinical context is limitation because it is not an endogenous metabolite. Therefore, bilirubin and oleic acid were selected as the final key metabolites based on their biological plausibility and more stable estimates.
